# SU‐Eohyeol Pharmacopuncture Ameliorates Parkinson’s Disease–Associated Pain via the CB1 and PPARγ Pathways in an MPTP‐Induced Mouse Model

**DOI:** 10.1155/prm/3334432

**Published:** 2026-05-31

**Authors:** Jung Im Kim, Heerim Yeo, Yousang Jo, Hyungjun Kim, Sang-Min Park, No Soo Kim

**Affiliations:** ^1^ KM Convergence Research Division, Korea Institute of Oriental Medicine, Daejeon, 34054, South Korea, kiom.re.kr; ^2^ College of Pharmacy, Chungnam National University, Daejeon, 34134, South Korea, cnu.ac.kr; ^3^ KM Science Research Division, Korea Institute of Oriental Medicine, Daejeon, 34054, South Korea, kiom.re.kr

**Keywords:** CB1, pain, Parkinson’s disease, pharmacopuncture, PPARγ

## Abstract

Pain is a ubiquitous nonmotor symptom in Parkinson’s disease (PD), substantially impairing quality of life. Although pharmacological and surgical interventions provide partial relief, effective treatment for PD‐associated pain remains limited. Pharmacopuncture, an injection of herbal extracts into acupoints, has demonstrated potential in pain management. This study evaluated the analgesic and neuroprotective effects of SU‐Eohyeol pharmacopuncture (SUEHP) in a 1‐methyl‐4‐phenyl‐1,2,3,6‐tetrahydropyridine (MPTP)–induced PD mouse model. C57BL/6N male mice received repeated intraperitoneal injections of MPTP to induce PD‐related pain. SUEHP was bilaterally administered at the GB34 acupoint or a control site. Pain sensitivity and motor function were evaluated, followed by postsacrifice histological assessments. Administration of SUEHP at GB34 significantly attenuated MPTP‐induced mechanical hypersensitivity and spinal c‐Fos expression, enhanced tyrosine hydroxylase and brain‐derived neurotrophic factor expression in the substantia nigra, and partially restored motor function. However, cotreatment with a CB1 or PPARγ antagonist eliminated these analgesic and neuroprotective effects, suggesting that SUEHP mediates its effects through specific pathways. Transcriptomic profiling of the midbrain and spinal cord revealed that SUEHP modulated inflammation, dopaminergic neurogenesis, and synaptic signaling pathways, which were typically reversed using cotreatment with CB1 or PPARγ antagonist. Collectively, SUEHP alleviates PD‐associated pain and neurodegeneration through CB1 and PPARγ pathways, suggesting its potential as a safe, noninvasive complementary therapy for PD symptom management.

## 1. Introduction

Parkinson’s disease (PD) is a progressive neurodegenerative disorder, and its prevalence is rapidly increasing following the improvement in human life expectancy [1]. PD is typically characterized by motor symptoms such as tremors, rigidity, and postural instability and is also accompanied by nonmotor symptoms, including sleep disturbances, depression, constipation, and pain [2, 3]. Pain affects approximately 40%–85% of patients with PD [4, 5] and is associated with the basal ganglia involved in pain processing; this is due to damage to the nigrostriatal system and is believed to involve complex neurobiological pathways [6].

Musculoskeletal pain, which is prevalent in patients with PD, results from gait disturbances, rigidity, and muscle and joint spasms associated with postural abnormalities [7]. This type of pain manifests in various body regions, including the spine, ankles, hips, shoulders, and neck, often causing severe discomfort due to uncontrollable muscle contractions [7, 8]. Current treatment strategies for pain management include physical exercise and administration of levodopa [9, 10]. Additionally, deep brain stimulation (DBS) [11, 12], nonsteroidal anti‐inflammatory drugs [13], and analgesics have been used to alleviate PD‐related pain [14]. However, for peripheral and neuromuscular pain, which is associated with sensory abnormalities such as numbness and paresthesia, the underlying mechanisms remain poorly understood, and treatment is typically based on approaches used for neuropathic pain [15]. Given that pain management is closely linked to patients’ quality of life, a comprehensive understanding of PD‐related pain and the development of effective therapeutic strategies are imperative [16].

Acupuncture, a traditional therapeutic approach widely used in East Asian medicine, has emerged as a promising nonpharmacological pain management strategy for PD‐related pain [17, 18]. In acupuncture, the selection of acupoints is based on neuroanatomical and neurophysiological principles [19]. The GB34 acupoint (Yanglingquan), chosen in this study, is located near the fibular head, where the common peroneal nerve converges with a dense vascular network. Unlike conventional intramuscular injections into the gluteal region, stimulation at GB34 influences neural circuits associated with cognition, motor control, and default mode networks [20]. Moreover, peripheral stimulation at GB34 activates melanin‐concentrating hormone (MCH) neurons in the lateral hypothalamus via afferent neural pathways [21]. Pharmacopuncture, which involves the direct injection of medicinal substances into acupoints following the meridian system of traditional medicine, provides analgesic and anti‐inflammatory effects [22, 23]. Moreover, pharmacopuncture enhances drug absorption efficiency and has been applied in the treatment of neurological and musculoskeletal disorders [24–26].

Jungsongouhyul pharmacopuncture (JSOHP) has demonstrated neuroregenerative and neuroprotective effects [27–29] and has exhibited potential benefits in managing PD‐related chest pain [30], acute traumatic shoulder pain [31], and lower back pain [32]. Therefore, we hypothesized that SU‐Eohyeol pharmacopuncture (SUEHP), a modified pharmacopuncture formulation incorporating deer antler extract into JSOHP, may serve as an alternative therapeutic strategy for pain modulation and motor symptom improvement in PD; deer antler extract exhibits anti‐inflammatory [33, 34], antioxidant [35], and antiaging properties [36]. This study aimed to determine whether SUEHP alleviates pain responses in an MPTP‐induced PD animal model and to provide experimental evidence supporting SUEHP’s potential as a complementary and alternative approach for pain management in PD.

## 2. Materials and Methods

### 2.1. Chemicals and SUEHP

SUEHP (lot NK0721003) was manufactured in accordance with the Korean Good Manufacturing Practices at the External Herbal Dispensary of Namsangcheon Korean Medicine Clinic (Yongin, Republic of Korea). The detailed prescription and production process for SUEHP has been previously described [37, 38]. The chemical composition of SUEHP was analyzed using gas chromatography–mass spectrometry and summarized in our previous report [38]. Selective cannabinoid receptor 1 (CB1) antagonist, SR141716A (#0923), and peroxisome proliferator–activated receptor gamma (PPARγ) antagonist, T0070907 (#2301), were obtained from Tocris Bioscience (Bristol, UK). 1‐Methyl‐4‐phenyl‐1,2,3,6‐tetrahydropyridine (MPTP, M2690) and Alkamuls EL‐620 (HY‐Y1890) were obtained from Tokyo Chemical Industry (Tokyo, Japan) and MedChemExpress (Monmouth Junction, NJ, USA), respectively. Injectable sterile saline (0.9% sodium chloride) was obtained from Dai Han Pharm (Seoul, Republic of Korea). Molecular biology‐grade absolute ethanol (E7148), sucrose (S0389), avertin (T48402), 2‐methyl‐2‐butanol (#152463), bovine serum albumin (BSA; A3059), and Triton X‐100 (TX‐100; X100) were obtained from Sigma‐Aldrich (St. Louis, MO, USA).

### 2.2. Animals

Eight‐week‐old male C57BL/6N mice were obtained from Orient Bio (Seongnam, Republic of Korea) and housed under controlled environmental conditions, with a temperature of 23 ± 1°C, relative humidity of 50%, and a 12‐h light/dark cycle. The animals were provided with *ad libitum* access to water and standard laboratory chow (Purina Co., Seoul, Republic of Korea). All experimental protocols were performed in compliance with the guidelines of the Animal Care and Use Committee of the Korea Institute of Oriental Medicine (approval numbers: 23‐010, February 22, 2023; 23‐085, September 26, 2023).

### 2.3. Animal Model and Pharmacopuncture Treatment

**FIGURE 1:**
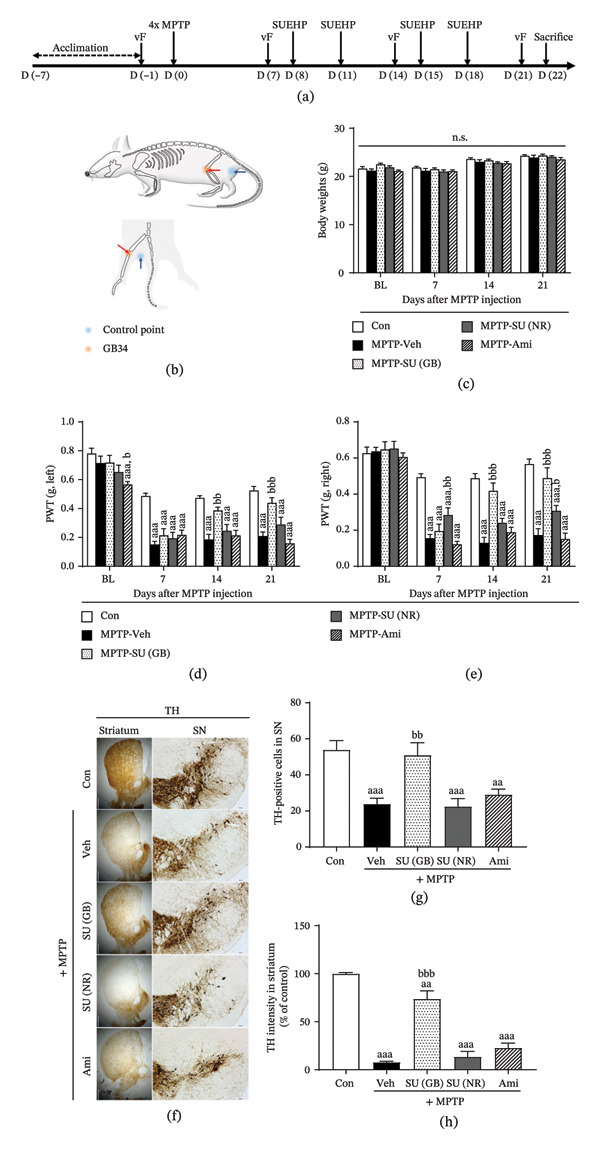
Effect of SUEHP on pain sensitivity and dopaminergic pathways in the MPTP‐induced PD animal model. (a) Experimental timeline for evaluating the effects of SUEHP in an MPTP‐induced PD mouse model. (b) SUEHP was administered at the GB34 acupoint, known for its efficacy in alleviating PD symptoms, with the gluteal region designated as the control point. Treatment was initiated 1 week after MPTP injection and was conducted twice weekly. (c) Body weight was monitored weekly throughout the experiment. Pain sensitivity was assessed using the von Frey filament test on (d) the left and (e) right hind paws, starting 7 days before MPTP administration and measured once a week until the end of the experiment. (f) After the experiment, the brain was removed and sectioned at 20 μm to examine the expression of tyrosine hydroxylase (TH) using DAB staining in the substantia nigra and striatum. (g) The number of TH‐positive cells in the substantia nigra was counted. (h) Intensity of TH staining in the striatum was quantified using ImageJ. Scale bar = 200 μm (substantia nigra) or 500 μm (striatum). Data are presented as the mean ± SEM (*n* = 10 except DAB staining, *n* = 5). ^aa^ *p* < 0.01, ^aaa^ *p* < 0.001 vs. Con. ^b^ *p* < 0.05, ^bb^ *p* < 0.01, ^bbb^ *p* < 0.001 vs. MPTP‐Veh. Abbreviations: BL, baseline; GB34, acupoint “Yanglingquan”; MPTP, 1‐methyl‐4‐phenyl‐1,2,3,6‐tetrahydropyridine; n.s., not significant; PD, Parkinson’s disease; PWT, paw withdrawal threshold; SEM, standard error of the means; SN, substantia nigra; SUEHP, SU‐Eohyeol pharmacopuncture; TH, tyrosine hydroxylase; vF, von Frey filament test. Experimental groups: Con, saline control + saline injection at GB34; MPTP‐Veh, MPTP + saline injection at GB34; MPTP‐SU(GB), MPTP + SUEHP injection at GB34; MPTP‐SU(NR), MPTP + SUEHP injection at a nonrelevant acupoint (gluteal region); MPTP‐Ami, MPTP + amitriptyline treatment.

Four injections of MPTP were administered intraperitoneally (10 mL/kg of body weight) at a dose of 20 mg/kg, every 2 h (Day 0). The control group received saline injections at the same frequency. One week after the final MPTP injection, SUEHP was administered twice a week until the end of the experiment, for a total of four injections (Figure 1a). SUEHP was injected bilaterally at GB34 and nonrelevant control acupoints (20 μL per acupoint) at a depth of 3 mm. GB34 and nonrelevant control acupoints were selected based on previous animal studies [39–41]. GB34, which has been traditionally used to treat movement disorders in East Asian medicine, is located on the lateral aspect of the lower leg, below the head of the fibula, in a palpable depression between the fibula and adjacent muscles. A nonrelevant control acupoint was located on the gluteus maximus 5 mm lateral from the tail base (Figure 1b). The control group for SUEHP received saline injections at GB34 under the same protocol. The positive control group for pain relief was administered intraperitoneal injections of amitriptyline dissolved in saline (10 mL/kg) at a dose of 10 mg/kg, instead of SUEHP, as described in a previous study [42].

### 2.4. Inhibitor Study

SR141716A (CB1 antagonist, 0.6 mg/mL) and T0070907 (PPARγ inhibitor, 1 mg/mL) were dissolved in a solvent mixture of ethanol, Alkamuls EL‐620%, and 0.9% saline in a 1:1:18 ratio [42, 43]. SR141716A (3 mg/kg) and T0070907 (5 mg/kg) were administered intraperitoneally (5 mL/kg of body weight) 30 min prior to SUEHP injection.

### 2.5. von Frey Filament Test

The von Frey filament test (Aesthesio, Danmic Global, San Jose, CA, USA) was used to determine the mechanical sensitivity of the hind paws of experimental animals. Mice were allowed to habituate in a mesh‐floored box for at least 30 min before the test. A von Frey filament of low bending force was applied to the center of the hind paw. Progressively higher bending forces (0.02, 0.04, 0.07, 0.16, 0.4, 0.6, 1, 1.4 g) were applied until one of the following pain‐related responses was observed: (1) sudden paw withdrawal, (2) prolonged paw withdrawal, or (3) licking of the paw. The bending force of the filament that triggered a pain‐related response was recorded as the nociceptive threshold, reflecting sensitivity to tactile stimuli.

### 2.6. Open Field Test

The open field test was used to assess the locomotor activity of experimental animals. The mice were initially placed at the center of a square area (40 × 40 × 40 cm) and allowed to move freely for 10 min. The total distance moved and the time spent in the central zone (24 × 24 cm) were recorded as primary measurements using video tracking software (HVS Image, Bicester, UK).

### 2.7. Rotarod Test

The rotarod test (ROTA‐ROD, B. S Technolab Inc., Seoul, Republic of Korea) was used to evaluate the coordinated motor function of mice and their ability to maintain balance on a rotating rod. The speed was increased from 4 to 40 rpm, with a 1‐rpm increment every 8.3 s. The test was repeated three times with a maximum duration of 300 s. The time each mouse remained on the accelerating rotating rod was used as the evaluation metric; those staying on the rod until the end of the test were counted as having reached the maximum duration.

### 2.8. Tissue Preparation

At the end of the experiment, the animals were anesthetized using an intraperitoneal injection of avertin (20 mL/kg body weight) at a concentration of 20 mg/mL. The brain and spinal cord intended for immunohistochemistry and immunofluorescence staining were dissected after sequential perfusion with phosphate‐buffered saline (PBS; pH 7.4) (#10010023; Thermo Fisher Scientific, Waltham, MA, USA) to remove residual blood and impurities, followed by 4% neutral‐buffered paraformaldehyde (P2031, Biosesang, Yongin, Republic of Korea) solution for tissue fixation. After dissection, the tissues were stored in 4% paraformaldehyde at 4°C for 48 h and then gradually dehydrated by immersion in a 30% sucrose solution for 4–5 days. The brain and spinal cord tissues for mRNA‐sequencing were extracted without any processing and were stored at −70°C until further analyses.

### 2.9. 3,3′‐Diaminobenzidine (DAB) Staining

Brain tissue sections with a thickness of 20 μm were prepared using a cryostat (CM1950; Leica Biosystems, Wetzlar, Germany). To reduce nonspecific binding, the tissue was incubated with 3% hydrogen peroxide (#88597, Sigma‐Aldrich) for 15 min. The tissue was then incubated for 1 h in a blocking solution (5% BSA in 0.1% TX‐100). Subsequently, the tissue was incubated overnight at 4°C with rabbit anti–tyrosine hydroxylase (TH) antibody (AB152; Sigma‐Aldrich) diluted in antibody diluent solution (2% BSA in 0.1% TX‐100). The following day, the tissue was incubated for 2 h with horseradish peroxidase‐conjugated goat anti‐rabbit secondary antibody (K4003; Agilent Technologies, Santa Clara, CA, USA) diluted in antibody diluent solution. After this incubation, the tissue was treated with ABC solution (PK‐6101, Vector Laboratories, Newark, CA, USA) according to the manufacturer’s instructions for 1 h. DAB (K3468; Agilent Technologies) staining was then performed. All washes were performed three times with PBS, for 5 min each time. All procedures, except for the primary antibody incubation, were performed at 22°C–24°C.

### 2.10. Immunofluorescence Staining

Brain tissue sections (20‐μm thickness) were prepared using a cryostat. To reduce nonspecific binding, the tissue was incubated with 3% hydrogen peroxide for 15 min. Subsequently, the tissue was incubated for 1 h in a blocking solution. Following this, the tissue was incubated overnight at 4°C with the primary antibody diluted using 3% BSA in 0.3% TX‐100. The primary antibodies used in this study were rabbit anti‐c‐Fos (ab190289; Abcam, Cambridge, UK), rabbit anti‐phosphorylated p38 mitogen‐activated protein kinase (p‐p38; 4511S; Cell Signaling Technology, Danvers, MA, USA), mouse anti‐cluster of differentiation 11b (CD11b; MA5‐17857; Thermo Fisher Scientific), rabbit anti‐glial fibrillary acidic protein (GFAP; #12389; Cell Signaling Technology), mouse anti‐neuronal nuclei (NeuN; #94403; Cell Signaling Technology), and rabbit anti‐brain‐derived neurotrophic factor (BDNF; ab108319; Abcam). The next day, the tissue was incubated for 1 h with secondary antibody, diluted using 3% BSA in 0.3% TX‐100. The secondary antibodies employed included goat anti‐mouse IgG‐Alexa Fluor 488 (A11001), donkey anti‐rabbit IgG‐Alexa Fluor 488 (A21206), and goat anti‐rabbit IgG‐Alexa Fluor 568 (A11036). After incubation, the tissue was treated with 4′,6‐diamidino‐2‐phenylindole (DAPI) solution (D1306) for 10 min. All secondary antibodies and DAPI were obtained from Thermo Fisher Scientific. All washes were performed three times with PBS, each lasting 5 min, and this sequence was repeated three times between each procedure. All procedures, except for the primary antibody incubation, were performed at 22°C–24°C.

### 2.11. *In Silico* Target Prediction of SUEHP

Potential protein targets of SUEHP were predicted based on its composition data and publicly available databases. Among the compounds in SUEHP, active compounds were selected based on two criteria—presence in known SUEHP ingredients and blood–brain barrier (BBB) permeability. Compound‐ingredient evidence was acquired from integrated natural product database, NPASS [44], and PubMed text mining. BBB permeability (logBB ≥ −1) of each compound was predicted by an *in silico* prediction method, LogBB_Pred [45]. Protein targets of SUEHP were predicted by obtaining the binding targets of active compounds. Binding targets were obtained from known targets in the pharmacological database, DrugBank [46], or from potent binding partners in high‐throughput screening assay of the publicly available screening database, PubChem BioAssays [47].

### 2.12. mRNA Sequencing

Total RNA was prepared from the spinal cord or midbrain using the Easy‐Spin Total RNA Extraction Kit (#17211, iNtRON Biotechnologies, Seongnam, Republic of Korea). In brief, the frozen tissues (10–100 mg) were suspended in the lysis buffer and mechanically homogenized using the Precellys 24 (Bertin Technologies, Montigny‐le‐Bretonneux, France). Then, the homogenized tissue lysates were processed according to the manufacturer’s instructions. RNA concentration was determined using the Qubit (Thermo Fisher Scientific), and its quality was determined using the Agilent 4200 TapeStation System (Agilent, Santa Clara, CA, USA). Criteria for determining RNA integrity for mRNA‐sequencing analysis were as follows: 28 s/18 s rRNA ratio > 1.0 and RNA integrity number > 7.0.

Total RNA was processed into sequencing libraries using the CORALL RNA‐Seq V2 Kit (Lexogen, Vienna, Austria). Polyadenylated RNA was enriched using the Poly(A) Selection Kit (Lexogen). Following cDNA synthesis and fragmentation, indexing was performed using Illumina indices (1–12), and libraries were enriched using polymerase chain reaction (PCR). Fragment sizes were evaluated using the Agilent TapeStation 4200 or 2100 Bioanalyzer. Library concentrations were quantified using quantitative PCR using the StepOne System (Life Technologies, Carlsbad, CA, USA). Sequencing was performed on an Illumina NovaSeq 6000 with 100‐bp paired‐end reads (Illumina, San Diego, CA, USA).

FastQC (https://www.bioinformatics.babraham.ac.uk/projects/fastqc/) was used for initial quality control. Adapter trimming and quality filtering were performed using the Fastp tool [48]. Cleaned reads were aligned to the reference genome using STAR [49], and transcript quantification was carried out with Salmon [50]. Read counts were normalized using the TMM + CPM method with the Python package conorm (https://github.com/amkatrutsa/conorm). Further data mining and visualization were performed using ExDEGA (eBiogen Inc., Seoul, Republic of Korea).

### 2.13. Statistics

All statistical analyses were performed using GraphPad Prism (v7.05, GraphPad Software, San Diego, CA, USA). Data are presented as the mean ± standard error of the mean (SEM). Group means were analyzed using the one‐way analysis of variance followed by Fisher’s least significant difference test for multiple comparisons. A *p*‐value of < 0.05 was considered statistically significant.

## 3. Results

### 3.1. SUEHP Attenuates MPTP‐Induced Pain Hypersensitivity and Dopaminergic Neuronal Deficits

To induce PD‐related pain, mice received intraperitoneal MPTP injections, and the von Frey filament test was performed according to the experimental timeline (Supporting Figure S1a). The results showed that a significant increase in pain sensitivity was observed in mice receiving MPTP on both foot pads, which lasted over 21 days (Supporting Figures S1b and S1c). Additionally, to examine the dopamine pathway through the nigrostriatal system, which is associated with PD progression, we assessed the expression of TH—the enzyme that converts tyrosine into L‐DOPA, the precursor of dopamine—in the substantia nigra and striatum. The results showed a significant decrease in TH expression in the substantia nigra (Supporting Figures S1d and S1e) and striatum (Supporting Figures S1f and S1g). Based on these findings, SUEHP was administered twice weekly, starting 1 week after MPTP injection (Figure 1a). The von Frey filament test was conducted to assess pain sensitivity prior to sacrifice. Acupoint injection was performed bilaterally at GB34, an acupoint known for pain relief in PD models [39–41], and a control point in the gluteal region, which is unrelated to GB34 (Figure 1b). Body weight did not show any significant differences among groups (Figure 1c). Amitriptyline, a commonly used neuropathic pain medication, was used as a positive control to compare the efficacy of SUEHP. Pain sensitivity assessments revealed that MPTP administration led to an increase in pain sensitivity as measured by the von Frey filament test. However, SUEHP treatment at the GB34 acupoint significantly reduced pain sensitivity, whereas SUEHP treatment at the control point and amitriptyline administration did not result in significant pain reduction (Figure 1d and e). The effect of SUEHP on the dopaminergic pathway was examined using TH immunostaining. The number of TH‐positive cells was quantified in the substantia nigra, and TH density was measured in the striatum. Compared to the control group, MPTP administration significantly reduced TH expression in both regions. However, SUEHP treatment at GB34 mitigated MPTP‐mediated reduction of TH expression, whereas SUEHP treatment at the control point and administration of amitriptyline did not produce significant changes (Figure 1f–h).

### 3.2. SUEHP Alleviates MPTP‐Induced Spinal c‐Fos Expression

**FIGURE 2:**
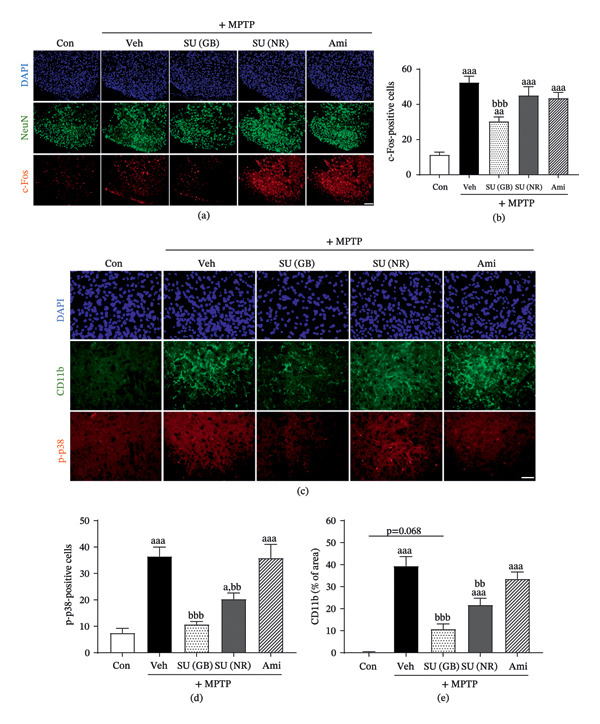
Effect of SUEHP on neuronal activation and microglial activation in the spinal cord of MPTP‐induced PD animals. (a) Pain signal transmission was examined in the dorsal horn of the spinal cord (L3–L6) using immunofluorescence staining for c‐Fos. (b) The number of c‐Fos‐positive cells was quantified. (c) Microglial activation was assessed in the spinal dorsal horn. (d) p‐p38‐positive cells were quantified, and (e) microglial activation was assessed by measuring the CD11b‐stained area. Data are presented as the mean ± SEM (*n* = 5). ^aaa^ *p* < 0.001 vs. Con. ^bb^ *p* < 0.01, ^bbb^ *p* < 0.001 vs. MPTP‐Veh. Scale bar = 100 μm (c‐fos) or 50 μm (p‐p38). Abbreviations: CD11b, cluster of differentiation 11b; DAPI, 4′,6‐diamidino‐2‐phenylindole; GB34, acupoint “Yanglingquan”; MPTP, 1‐methyl‐4‐phenyl‐1,2,3,6‐tetrahydropyridine; NeuN, neuronal nuclei; PD, Parkinson’s disease; p‐p38, phosphorylated p38 mitogen‐activated protein kinase; SEM, standard error of the mean; SUEHP, SU‐Eohyeol pharmacopuncture. Experimental groups: Con, saline control + saline injection at GB34; MPTP‐Veh, MPTP + saline injection at GB34; MPTP‐SU(GB), MPTP + SUEHP injection at GB34; MPTP‐SU(NR), MPTP + SUEHP injection at a nonrelevant acupoint (gluteal region); MPTP‐Ami, MPTP + amitriptyline treatment.

We assessed the expression of c‐Fos, a marker of neuronal activation in pain pathways, in the dorsal horns of the L3–L6 spinal cord region. Compared to the control group, MPTP administration significantly increased c‐Fos expression in the spinal cord. Notably, SUEHP treatment at GB34 mitigated this MPTP‐induced increase in c‐Fos expression, whereas SUEHP treatment at the control point or amitriptyline administration had no significant effect (Figure 2a and b). These findings, consistent with the results of the pain sensitivity tests, demonstrate that SUEHP treatment at GB34 alleviates pain hypersensitivity in an MPTP‐induced PD model and reduces pain transmission signals at the molecular level.

### 3.3. SUEHP Attenuates MPTP‐Induced Inflammatory Responses

We assessed p‐p38, a key marker involved in cellular stress and inflammatory signaling [51]. Following MPTP administration, p‐p38 levels were significantly elevated. SUEHP treatment at GB34 markedly reduced p‐p38 expression, while SUEHP treatment at the control point also resulted in a reduction, albeit to a lesser extent (Figure 2c and d).

Additionally, we assessed CD11b, a marker of astrocytes and microglia activation, which are the primary immune‐related cells in the central nervous system (CNS). MPTP administration led to increased activation of both cell types in the spinal cord. However, although SUEHP treatment did not alter astrocyte activation (Supporting Figures S2a and S2b), it significantly reduced microglial activation (Figure 2c and e). These findings suggest that the modulation of p‐p38‐associated pain signaling in the spinal cord following SUEHP treatment may be more associated with microglia than astrocytes. Notably, amitriptyline administration did not induce significant changes in inflammatory responses, indicating that its analgesic effects are not mediated through inflammation‐related mechanisms.

### 3.4. Data‐Driven *In Silico* Prediction of Potential Protein Targets of SUEHP

**FIGURE 3:**
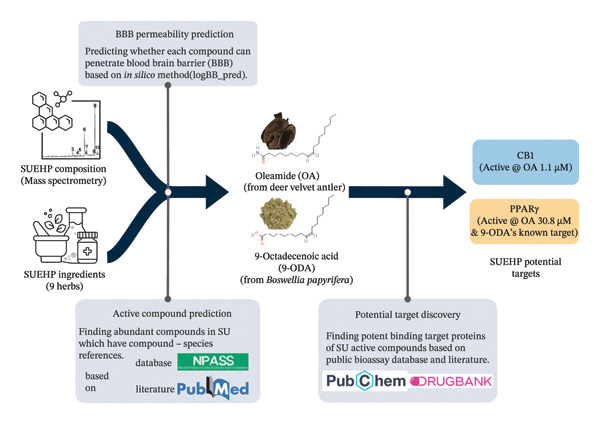
In silico screening procedure for the prediction of SUEHP potential protein targets from SUEHP composition and ingredients data. “Active @ ODA 1.1 μM” denotes that oleamide induces the response of a target at 1.1 μM concentration. Nine herbs used for SUEHP are as follows: Olibanum, Gardeniae Fructus, Myrrha, Persicae Semen, Corydalis Tuber, Salviae Miltiorrhizae Radix, Sappan Lignum, Paeoniae Radix, and deer antler extract. Abbreviations: BBB, blood–brain barrier; CB1, cannabinoid receptor 1; PPARγ, peroxisome proliferator–activated receptor gamma; SUEHP, SU‐Eohyeol pharmacopuncture.

To clarify the therapeutic mechanism of SUEHP, its potential protein targets should be determined. Using publicly available data, we predicted the potential protein targets of SUEHP using mass spectrometry composition data [38] and ingredient details (Figure 3). We first predicted active compounds of SUEHP before finding potential protein targets. The active compound is defined as an abundant compound of the SUEHP composition based on two criteria: It should be present in the SUEHP ingredients and should be capable of crossing the BBB. From SUEHP composition data, abundant compounds such as oleamide, 9‐octadecenoic acid, octadecane, cis‐vaccenic acid, eicosane, hexadecanamide, and oleyl alcohol were selected. Then, we investigated compound‐ingredient evidence of these compounds using the integrated natural product database. Among the abundant compounds in SUEHP, only oleamide and 9‐octadecenoic acid have evidence of being present in one of its ingredients, and both these compounds were confirmed to penetrate the BBB (logBB ≥ −1) using an *in silico* prediction method. Therefore, oleamide and 9‐octadecenoic acid were selected as SUEHP active compounds.

Next, we identified the potential protein targets of SUEHP by obtaining the binding targets of its active compounds. For oleamide, it induces a potent response with CB1 at 1.14 μM (PubChem AID238883) and an intermediate response with PPARγ at 30.8 μM (PubChem AID743191). For 9‐octadecenoic acid, its cis‐form has a known drug target, PPARγ (from DrugBank). From these results, CB1 or PPARγ was determined as potential binding target of SUEHP.

### 3.5. SUEHP Alleviates MPTP‐Induced Pain Responses via CB1 or PPARγ

**FIGURE 4:**
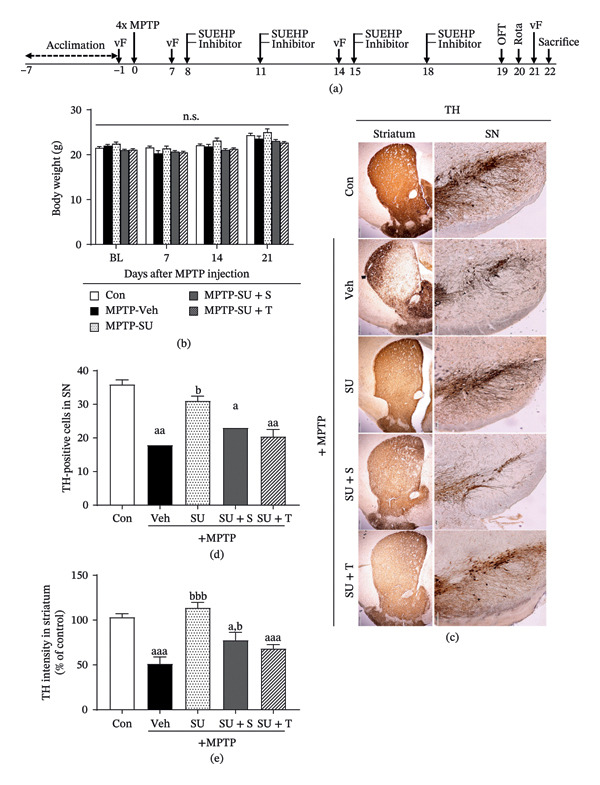
Effect of CB1 antagonist or PPARγ inhibitor on the SUEHP‐mediated recovery of dopamine pathways. (a) Experimental timeline for evaluating the effects of CB1 antagonist or PPARγ inhibitor in MPTP‐induced PD mouse model treated with SUEHP treatment at GB34 acupoint. CB1 antagonist or PPARγ inhibitor was administered intraperitoneally 30 min before SUEHP treatment at the GB34 acupoint. (b) Body weight was monitored weekly throughout the experiment. (c) After the experiment, the brain was extracted and sectioned at 20 μm to assess tyrosine hydroxylase (TH) expression using DAB staining in the substantia nigra and striatum. (d) The number of TH‐positive cells in the substantia nigra and (e) TH intensity in the striatum was determined using ImageJ. Scale bar = 200 μm (substantia nigra) or 500 μm (striatum). Data are presented as the mean ± SEM (*n* = 10 except for DAB staining, *n* = 4). ^aa^ *p* < 0.01, ^aaa^ *p* < 0.001 vs. Con. ^bb^ *p* < 0.01, ^bbb^ *p* < 0.001 vs. MPTP‐Veh. Abbreviations: BL, baseline: CB1, cannabinoid receptor 1; MPTP, 1‐methyl‐4‐phenyl‐1,2,3,6‐tetrahydropyridine; n.s., not significant; OFT, open field test; PD, Parkinson’s disease; PPARγ, peroxisome proliferator‐activated receptor gamma; SEM, standard error of the mean; SN, substantia nigra; SUEHP, SU‐Eohyeol pharmacopuncture; TH, tyrosine hydroxylase; vF, von Frey filament test. Experimental groups: Con, saline control + saline injection at GB34; MPTP‐Veh, MPTP + saline injection at GB34; MPTP‐SU, MPTP + SUEHP injection at GB34; MPTP‐SU + S, MPTP + SUEHP injection at GB35 with SR141716A pretreatment; MPTP‐SU + T, MPTP + SUEHP injection at GB34 with T0070907 pretreatment.

To determine whether CB1 and PPARγ, predicted as potential targets of SUEHP, are involved in SUEHP‐mediated pain modulation, animal studies were performed according to a timeline established based on previous studies (Figure 4a). Body weights remained constant throughout the experiment, irrespective of SUEHP, PPARγ inhibitor, or CB1 antagonist treatment (Figure 4b). To investigate changes in the dopaminergic pathway, TH immunostaining was performed in the substantia nigra and striatum (Figure 4c). Compared to the control group, TH levels decreased following MPTP administration but were restored in the group receiving SUEHP treatment at GB34. However, coadministration of T0070907 or SR141716A blocked TH recovery. This pattern was observed in both the substantia nigra (Figure 4d) and striatum (Figure 4e), although SUEHP treatment combined with SR141716A partially prevented TH depletion in the striatum. These findings suggest that SUEHP‐mediated TH recovery is likely regulated via pathways involving PPARγ or CB1.

**FIGURE 5:**
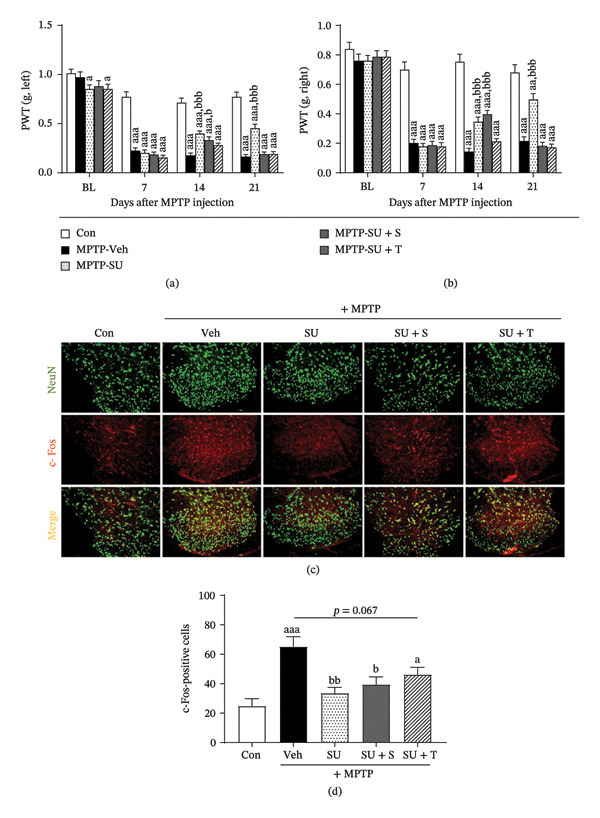
Effects of CB1 antagonist or PPARγ inhibitor on the SUEHP‐induced modulation of pain sensitivity and neural activation Pain sensitivity was assessed using the von Frey filament test on the (a) left and (b) right hind paws of PD animals following SUEHP treatment at the GB34 acupoint in the presence of CB1 antagonist (SR141716A) or PPARγ inhibitor (T0070907) cotreatment, starting 7 days before MPTP administration and measured once per week until the end of the experiment. (c) Pain signal transmission was evaluated in the dorsal horn of the spinal cord (L3–L6) using immunofluorescence staining for c‐Fos, and (d) the number of c‐Fos‐positive cells was quantified. Scale bar = 100 μm. Data are presented as the mean ± SEM (*n* = 10 except for c‐Fos staining, *n* = 4). ^a^ *p* < 0.05, ^aa^ *p* < 0.01, ^aaa^ *p* < 0.001 vs. Con. ^b^ *p* < 0.05, ^bb^ *p* < 0.01, ^bbb^ *p* < 0.001 vs. MPTP‐Veh. Abbreviations: BL, baseline; CB1, cannabinoid receptor 1; MPTP, 1‐methyl‐4‐phenyl‐1,2,3,6‐tetrahydropyridine; NeuN, neuronal nuclei; PD, Parkinson’s disease; PPARγ, peroxisome proliferator‐activated receptor gamma; SEM, standard error of the mean; SUEHP, SU‐Eohyeol pharmacopuncture; PWT, paw withdrawal threshold. Experimental groups: Con, saline control + saline injection at GB34; MPTP‐Veh, MPTP + saline injection at GB34; MPTP‐SU, MPTP + SUEHP injection at GB34; MPTP‐SU + S, MPTP + SUEHP injection at GB35 with SR141716A pretreatment; MPTP‐SU + T, MPTP + SUEHP injection at GB34 with T0070907 pretreatment.

PPARγ and CB1 are closely associated with pain signaling; hence, we further examined pain sensitivity. MPTP administration increased pain sensitivity in both hind paws; the pain was significantly relieved after SUEHP treatment at GB34. However, coadministration of SR141716A or T0070907 prevented this pain‐relieving effect, further supporting the role of PPARγ or CB1 in SUEHP‐mediated pain modulation (Figure 5a and b). Additionally, c‐Fos expression, a marker of neuronal activation in pain pathways, was analyzed using immunohistochemistry. MPTP‐induced c‐Fos expression was reduced by SUEHP treatment at GB34 acupoint. However, cotreatment with SR141716A or T0070907 attenuated this reduction, suggesting that SUEHP modulates pain transmission via CB1 or PPARγ‐related mechanisms (Figure 5c and d).

To further evaluate whether SUEHP treatment exerts beneficial effects on motor deficits in the MPTP‐induced PD animal model, we performed the open field and rotarod tests. In the open field test (Supporting Figure S3a), the total distance covered was reduced in the MPTP group; moreover, although SUEHP treatment tended to increase locomotor activity, the change did not reach statistical significance. Coadministration of SR141716A or T0070907 did not significantly alter this outcome (Supporting Figure S3b). Analysis of the time spent in the center zone revealed no significant differences among the experimental groups. However, cotreatment of SUEHP with T0070907 resulted in a reduction in the time spent in the center zone compared to the group treated with MPTP alone (Supporting Figure S3c). In the rotarod test, which measures the time animals remain on an accelerating rotating rod, the MPTP group showed a reduced latency to fall compared to the normal control group. This motor impairment was ameliorated by SUEHP treatment. The improvement in motor performance induced by SUEHP was eliminated by SR141716A, whereas T0070907 had no effect, suggesting that the motor benefits of SUEHP may be mediated through CB1 activation rather than PPARγ (Supporting Figure S3d).

### 3.6. SUEHP Restores MPTP‐Induced Reduction of Neurotrophic Factors

**FIGURE 6:**
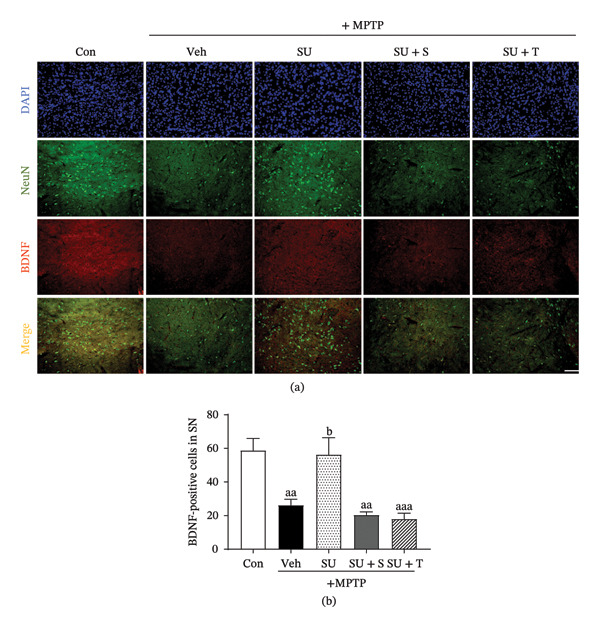
Effects of CB1 antagonist or PPARγ inhibitor treatment on the SUEHP‐mediated recovery of BDNF expression in the substantia nigra of the MPTP‐induced PD animal model. (a) Expression of BDNF, a neuroprotective factor, in the substantia nigra of the MPTP‐induced PD mouse model following SUEHP treatment at GB34 acupoint in the presence of CB1 antagonist (SR141716A) or PPARγ inhibitor (T0070907) cotreatment was determined using immunofluorescence staining. (b) The number of BDNF‐positive cells was quantified using ImageJ. Data are presented as the mean ± SEM (*n* = 4). ^aa^ *p* < 0.01, ^aaa^ *p* < 0.001 vs. Con. ^b^ *p* < 0.05 vs. MPTP‐Veh. Scale bar = 100 μm. Abbreviations: BDNF, brain‐derived neurotrophic factor; DAPI, 4′,6‐diamidino‐2‐phenylindole; MPTP, 1‐methyl‐4‐phenyl‐1,2,3,6‐tetrahydropyridine; NeuN, neuronal nuclei; PD, Parkinson’s disease; SEM, standard error of the mean; SN, substantia nigra; SUEHP, SU‐Eohyeol pharmacopuncture. Experimental groups: Con, saline control + saline injection at GB34; MPTP‐Veh, MPTP + saline injection at GB34; MPTP‐SU, MPTP + SUEHP injection at GB34; MPTP‐SU + S, MPTP + SUEHP injection at GB35 with SR141716A pretreatment; MPTP‐SU + T, MPTP + SUEHP injection at GB34 with T0070907 pretreatment.

BDNF plays a crucial role in neuronal survival, growth, development, synaptic plasticity, memory and learning, regulation of depression and anxiety, and neuroregeneration following brain injury [52]. Acupuncture at GB34 in PD animal models stimulates nerve conduction in MCH neurons, thereby transmitting signals to the substantia nigra and hippocampus [21]. Hence, we examined BDNF expression in these regions of PD animals following SUEHP treatment in the presence and absence of PPARγ inhibitor or CB1 antagonist (Figure 6a). In the substantia nigra, BDNF expression was significantly reduced following MPTP administration, which was restored to normal levels after SUEHP treatment at the GB34 acupoint. However, coadministration of a T0070907 or SR141716A inhibited SUEHP‐mediated BDNF recovery (Figure 6b). Similarly, compared to the control group, MPTP administration led to a reduction in BDNF levels across the hippocampal cornu ammonis (CA)1 (Supporting Figures S4a and S4b), CA3 (Supporting Figures S4c and S4d), and dentate gyrus (DG) regions (Supporting Figures S4e and S4f). SUEHP treatment at GB34 acupoint significantly increased BDNF expression in these areas. However, in contrast to the substantia nigra, coadministration of T0070907 or SR141716A did not significantly alter BDNF expression in the hippocampus. These findings suggest that SUEHP facilitates BDNF recovery in the substantia nigra through CB1‐ or PPARγ‐related pathways, whereas its effects in the hippocampus may involve alternative mechanisms.

### 3.7. SUEHP Alters MPTP‐Induced Transcriptomic Characteristics in the Midbrain and Spinal Cord

**FIGURE 7:**
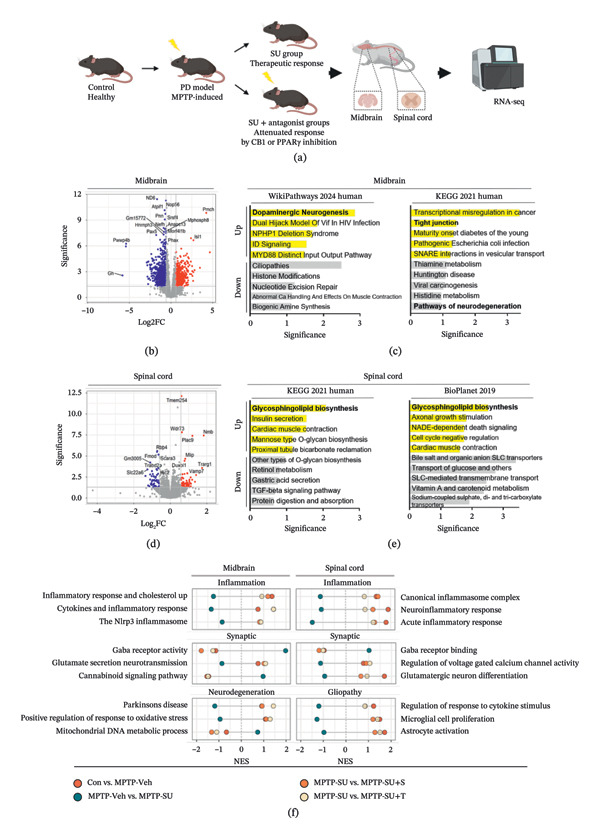
Transcriptomic analysis of the effects of SUEHP in the midbrain and spinal cord of MPTP‐treated mice. (a) Schematic overview of the experimental groups. Midbrain and spinal cord tissues were collected for transcriptomic analysis. (b, c) Volcano plots of DEGs in the midbrain and spinal cord, respectively. Genes with fold change > 1.5 and adjusted *p* < 0.05 are indicated in red (upregulated) or blue (downregulated). Significance is represented as −log_10_ (adjusted *p*). (d, e) ORA of upregulated (yellow) and downregulated (gray) DEGs was performed using EnrichR with the WikiPathways 2024 Human, KEGG 2021 Human, and BioPlanet 2019 databases. Top five enriched pathways for each direction are shown for the midbrain and spinal cord, respectively. The significance of enrichment was quantified as −log10 (*p*‐value). (f) GSEA summarizes functional categories: inflammation, synaptic, and neurodegeneration modules in the midbrain and inflammation, synaptic, and gliopathy modules in the spinal cord. Bubble size reflects −log_10_(FDR), and horizontal position indicates the NES. Abbreviations: DEG, differentially expressed gene; GSEA, gene set enrichment analysis; NES, normalized enrichment score; ORA, over‐representation analysis; experimental groups: Con, saline control + saline injection at GB34; MPTP‐Veh, MPTP + saline injection at GB34; MPTP‐SU, MPTP + SUEHP injection at GB34; MPTP‐SU + S, MPTP + SUEHP injection at GB35 with SR141716A pretreatment; MPTP‐SU + T, MPTP + SUEHP injection at GB34 with T0070907 pretreatment.

To investigate the global transcriptomic effects of SUEHP, we performed mRNA‐sequencing of the midbrain and spinal cord across five experimental groups: Control, MPTP + vehicle, MPTP + SUEHP, and MPTP + SUEHP coadministered with either SR141716A or T0070907 (Figure 7a). SUEHP treatment in MPTP mice induced broad transcriptomics alterations, with 2525 differentially expressed genes (DEGs) identified in the midbrain (935 upregulated and 1590 downregulated, Figure 7b) and 111 in the spinal cord (61 upregulated and 50 downregulated, Figure 7c), compared to the MPTP + vehicle group. Pathway enrichment analysis based on the identified DEGs revealed biological pathways that support both the pathological changes induced by MPTP and the therapeutic effects of SUEHP. In the midbrain, SUEHP treatment was associated with upregulation of genes enriched in pathways involved in “dopaminergic neurogenesis” and “tight junction” (Figure 7d). In contrast, downregulated genes were abundant in pathways associated with “neurodegenerative processes.” In the spinal cord, where pain signaling is prominent, genes upregulated by SUEHP were abundant in pathways related to “glycosphingolipid biosynthesis” (Figure 7e), a lipid metabolic process linked to PPARγ‐regulated signaling networks.

We hypothesized that the analgesic effects of SUEHP are mediated through CB1 or PPARγ‐related mechanisms; hence, we next investigated whether cotreatment with their respective antagonists (SR141716A or T0070907) could reverse the transcriptomic changes induced by SUEHP (Figure 7f). Gene set enrichment analysis (GSEA) revealed that SUEHP suppressed inflammatory responses in both the midbrain and spinal cord, consistent with our experimental observations, whereas these effects were typically reversed by coadministration of the antagonists. In the midbrain, SUEHP promoted inhibitory neurotransmission by enhancing “gamma‐aminobutyric acid (GABA) receptor activity” and “cannabinoid signaling” and decreasing “glutamate secretion.” It also downregulated neurodegeneration‐related pathways, including that for “PD,” “positive regulation of response to oxidative stress,” and “mitochondrial DNA metabolic process.” In the spinal cord, SUEHP modulated synaptic signaling by downregulating “regulation of voltage‐gated calcium channel activity” and “regulation of neurotrophin Trk receptor signaling pathway” and upregulating “GABA receptor binding,” indicating involvement in nociceptive transmission. Furthermore, it suppressed “microglial proliferation,” “astrocyte activation,” and “regulation of response to cytokine stimulus,” contributing to the reduction of proinflammatory factors that drive neuroinflammation. These transcriptomic changes were reversed by CB1 or PPARγ inhibition.

## 4. Discussion

In modern medicine, PD remains an intractable neurodegenerative disorder that lacks a clinical cure. Therefore, slowing the progression of the disease and managing symptoms is crucial to effectively maintain the patient’s quality of life [53, 54]. Among the various symptoms of PD, pain is a significant factor that directly affects daily life [55]. However, direct therapeutic intervention for PD‐associated pain is currently unavailable.

In traditional Chinese medicine, acupuncture has been used as an effective treatment for pain management [56, 57] and alleviates various symptoms such as cognitive impairment [58], anxiety [59], and sleep disturbances [60] in patients with PD. In our study, pharmacopuncture administered at GB34 demonstrated greater efficacy in mitigating PD‐related symptoms—such as dopaminergic neuronal suppression induced by MPTP, motor dysfunction, inflammation, and pain responses—compared to intragluteal injection. This observation suggests that the therapeutic outcome of pharmacopuncture may vary significantly depending on the site of administration. Unlike intramuscular injection into the gluteus maximus, which facilitates systemic drug distribution, acupoints such as GB34, which are selected based on neuroanatomical significance [19], offer more direct access to peripheral nerve bundles and reveal the potential to activate specific neural pathways. In neurodegenerative conditions such as PD, targeted acupoint administration may therefore yield superior therapeutic outcomes. Additionally, drug delivery via acupoints could minimize interference from the BBB and instead rely on peripheral nerve fiber stimulation to propagate therapeutic signals through the spinal cord and brainstem to central neural circuits. Concurrently, site‐specific acupoint injection is more effective than nonspecific intramuscular injection in clinical settings, suggesting that the injection site plays a critical role in determining drug distribution and receptor engagement [61]. In our study, pharmacopuncture at the GB34 acupoint was more effective than gluteal injection in modulating central PD pathology, enhancing the survival of dopaminergic neurons, and attenuating neuroinflammation. Amitriptyline, which was used as a positive control in this study, is widely recognized as a first‐line pharmacotherapy for neuropathic pain [62], but it did not exert a significant analgesic effect in MPTP‐induced pain. MPTP‐induced pain is expected to induce rigidity‐associated musculoskeletal pain and has been reported to increase hyperalgesia and allodynia [63, 64]. In addition, aberrant pain modulation is influenced by multiple factors, including degeneration of sensory neuronal populations within the dorsal horn of the spinal cord [65, 66] and astrocyte‐mediated activation of proinflammatory signaling pathways [66]. Accordingly, amitriptyline may not have been an appropriate positive control in this study, as its analgesic effects are primarily mediated through inhibition of noradrenaline reuptake within the dorsal horn [67]. Therefore, future studies should consider the use of dopaminergic agents targeting dopamine deficiency as more appropriate positive control drugs.

Stimulation of GB34 reduces dopaminergic depletion, oxidative stress, and inflammation, while simultaneously enhancing neuroprotective molecular responses in PD animal models [68–70]. Furthermore, GB34 stimulation may improve motor and cognitive deficits in PD through the activation of MCH neurons, which project to the substantia nigra and hippocampus [21]. These findings highlight the potential of peripheral nerve stimulation in modulating motor and nonmotor symptoms of PD [21]. Our study hypothesized that SUEHP treatment, an advanced form of acupuncture that combines mechanical acupoint stimulation with pharmacological administration, could have beneficial effects on PD‐associated pain. Using an MPTP‐induced pain animal model, we observed that SUEHP significantly reduced sensitivity to mechanical stimulus and pain‐associated molecular signaling. In contrast, behavioral assessments of locomotor activity, coordinated motor function, and the ability to maintain balance indicated that MPTP‐induced motor deficits were not significantly improved by SUEHP. This finding suggests that, although the dopaminergic system may have been partially restored by SUEHP, this restoration may not have been sufficient to normalize motor function. One possible explanation is that motor function is not determined exclusively by TH expression levels, but rather by multiple aspects of neural circuit dynamics, including alterations in dopamine‐dependent striatal synaptic plasticity [71] and changes in basal ganglia circuitry [72]. Accordingly, SUEHP may have preferentially affected sensory processing, such as pain modulation, rather than motor function.

Pain transmission involves multiple complex pathways, one of which is the CB receptor signaling pathway [73]. CB‐based therapeutics, such as cannabidiol (CBD), regulate dopaminergic signaling [74], anxiety [75], addiction [76], and schizophrenia‐related symptoms [77] via CB1 and PPARγ activation. Additionally, CBD and PPARγ modulate dopaminergic activity in the mesocorticolimbic system, thereby influencing neuropsychiatric symptoms [74, 78]. PPARγ activation has demonstrated anti‐inflammatory effects by inhibiting inflammatory cytokine release and amyloid‐beta accumulation in AD models [79, 80], suggesting that PPARγ‐mediated modulation using CBD may have therapeutic potential in PD. Although CBD possesses substantial therapeutic potential as an anticonvulsant, anxiolytic, and antipsychotic agent, its clinical application has been associated with various adverse effects in patients with epilepsy and psychiatric disorders, including fatigue, somnolence, diarrhea, vomiting, and hepatic dysfunction [81], Moreover, preclinical studies have reported hepatocellular injury, reproductive toxicity, CNS depression, and neurotoxicity [81], In the present study, SUEHP—comprising oleamide, an endogenous CB1 receptor agonist, and 9‐octadecenoic acid, a PPARγ agonist—as its principal bioactive constituents [38], was locally administered at GB34. This approach elicits synergistic activation of CB1 and PPARγ pathways, potentially conferring therapeutic benefits while minimizing the systemic adverse effects typically observed with CBD‐based compounds.

Through data‐driven *in silico* predictions, we identified oleamide and 9‐octadecenoic acid, two major constituents of SUEHP formulation, as potential ligands for CB1 and PPARγ, which are involved in pain‐related signaling. To experimentally validate these findings, we administered a PPARγ inhibitor or a CB1 antagonist prior to SUEHP treatment. The results indicated that blocking CB1 or PPARγ significantly attenuated the pain‐sensitization‐reducing effects of SUEHP. However, given the complexity of pain signaling pathways, the results for c‐Fos expression in the spinal cord were not entirely conclusive. Nevertheless, these findings suggest that SUEHP modulates pain responses in PD‐induced animal models via CB1 or PPARγ pathways.

Additionally, we investigated the neuroprotective effects of GB34‐targeted SUEHP on the substantia nigra and hippocampus. The results revealed that BDNF, which was previously diminished in PD models, was significantly restored in both brain regions following SUEHP treatment. This finding suggests that SUEHP may regulate pain‐related signaling and prevent dopaminergic neuronal loss through neuroprotective mechanisms that were confirmed in this study. However, the precise molecular mechanism underlying the neuroprotective potential of SUEHP should be further investigated to support its clinical use for PD‐associated pain.

In addition, the anterior cingulate cortex (ACC) and periaqueductal gray (PAG), which are critically involved in the cognitive and affective dimensions of pain, have been implicated in the dysfunction of pain processing systems in PD [82, 83]; therefore, it is necessary to investigate the effects of SUEHP on these regions. The ACC influences inflammatory pain modulation through regulation of CB1 receptor activation [84], while activation of CB1 receptors in the PAG exerts analgesic effects via bidirectional modulation of GABAergic and glutamatergic neuronal activity [85]. Furthermore, pain modulation mediated by PPAR signaling pathways in the ACC and PAG can also be considered [86, 87]. Therefore, if SUEHP exerts analgesic effects through CB1 or PPARγ‐dependent pathways, it is likely to influence the ACC and/or PAG, warranting further study.

Given that PD is caused by a dysfunction of the nigrostriatal pathway owing to degeneration of dopaminergic neurons in the substantia nigra, the enrichment of dopaminergic neurogenesis in the midbrain following SUEHP treatment is a crucial observation. Among the diverse cell types comprising the CNS, microglia play a dual role by mediating neuroinflammatory responses through neuroprotective mechanisms and are particularly implicated in chronic neuroinflammation, which is closely associated with neurotoxicity and neurodegeneration [88]. In PD, microglial activation contributes to dopaminergic neuronal degeneration, accompanied by increased levels of proinflammatory cytokines and decreased BDNF levels [89].

In this study, we demonstrated that SUEHP treatment suppressed microglial activation and reduced p‐p38 levels in the spinal cord, a key region involved in nociceptive signaling, thereby alleviating neuroinflammatory responses. Consistent with these findings, GSEA involving “inflammation” confirmed that SUEHP treatment attenuated inflammatory responses in both the midbrain and spinal cord. Furthermore, considering that the integrity and survival of dopaminergic neurons are crucial factors in the pathogenesis of PD, experimental analyses showed that SUEHP treatment increased the expression of BDNF in both the substantia nigra and hippocampus and effectively prevented the loss of dopaminergic neuronal integrity. In line with these results, transcriptomic analysis revealed that Enrichr‐based profiling of the midbrain showed an upregulation of “dopaminergic neurogenesis” and a downregulation of “pathways of neurodegeneration.” Additionally, a summary of the functional categories within the “neurodegeneration” module confirmed a reduced association with “PD,” thereby providing further evidence supporting the neuroprotective effects of SUEHP. However, although SUEHP treatment increased BDNF expression in the SN and hippocampus, pharmacological inhibition of CB1 or PPARγ was less effective in suppressing BDNF expression in the hippocampus than in the SN. These differential effects are likely attributable to region‐specific differences in cellular composition, neurotransmitter systems, receptor expression patterns, and mechanisms regulating BDNF expression. While BDNF in the SN is implicated in dopaminergic neuronal protection [90] and modulation of microglia‐mediated inflammatory responses [91], BDNF in the hippocampus regulates long‐term synaptic plasticity through glutamatergic neuronal networks [92]. Accordingly, the dependence on CB1 or PPARγ signaling may differ between the SN and hippocampus.

Disruption of tight junction integrity is a contributing factor in the onset and progression of PD [93–95]; moreover, based on transcriptomic analysis, the observed enhancement of tight junction‐related pathways following SUEHP treatment may represent a noteworthy finding. Additionally, enrichment analysis of upregulated genes in the spinal cord revealed alterations in “glycosphingolipid biosynthesis,” which may be associated with dysregulated lipid metabolism observed in PD [96]. Notably, SUEHP may possess the potential to modulate nociceptive signaling through mechanisms involving CB1 receptors or PPARγ, which are closely linked to lipid metabolic pathways [97, 98]. According to previous reports, glycosphingolipids synthesized from ceramide via glucosylceramide directly interact with the A/B domain of PPARγ, thereby enhancing its activation [99].

Moreover, the activation of c‐Fos in spinal GABAergic neurons has been well‐documented in animal models exposed to acute or chronic pain stimuli and is closely associated with pain and inflammation [100]. In this study, we observed that SUEHP treatment reduced the elevated c‐Fos response in the spinal cord. Term analysis of “synaptic” processes in the midbrain or spinal cord also revealed increased GABA receptor activity or binding. GABA inhibits nociceptive transmission in both the brain and spinal cord [89]; hence, along with the transcriptomic data, these findings suggest that SUEHP treatment may alleviate MPTP‐induced pain and neuroinflammation and preserve the integrity of dopaminergic neurons in PD.

Although the present study suggests that SUEHP may potentially ameliorate pain and dopaminergic neurodegeneration in an MPTP‐induced mouse model of PD through several biological pathways, further studies are needed to fully characterize its analgesic effects. The prevalence of PD differs significantly by sex, with male sex exhibiting an incidence rate 1.5 to 2 times higher than that of female sex. Accordingly, male animals were prioritized in the PD animal model experiments. However, considering that PD also occurs in female sex, future studies should incorporate female animals to ensure a more comprehensive understanding of disease mechanisms and potential therapeutic interventions [101, 102]. Additionally, further investigation is required to elucidate the precise mechanisms involved. Considering the close association of tight junction integrity, lipid metabolism, and inflammatory responses with dopaminergic degeneration and PD‐related symptoms, further research into the multifaceted effects of SUEHP would be of significant value. Although this study does not fully elucidate the mechanisms underlying MPTP‐induced pain, the attenuation of pain‐related signaling by SUEHP may involve CB1‐ or PPARγ‐dependent mechanisms, providing promising evidence for its therapeutic potential.

## 5. Conclusions

This study has some limitations that need to be considered when interpreting the reliability and translational relevance of the preclinical findings. First, the exclusive use of male murine models may obscure sex‐specific differences in PD pathophysiology. Given the well‐documented neuroprotective effect of estrogen, further validation in female murine models is warranted. Second, although the *in silico* analyses employed in this study provided valuable predictions regarding receptor–ligand interactions, they cannot fully account for binding affinity, bioavailability, or pharmacokinetics *in vivo*, highlighting the need for empirical evidence to confirm the molecular targets. Lastly, while the MPTP‐induced acute PD murine model is widely used, it does not recapitulate the progressive nature of human PD. Therefore, the findings should be validated in alternative preclinical systems, such as α‐synuclein overexpression, to establish reproducibility and enhance translational relevance.

Collectively, this study provides preclinical evidence that SUEHP can modulate pain through the CB1 or PPARγ pathway, positioning them as potential therapeutic targets for pain modulation in PD. Furthermore, our findings suggest that SUEHP enhances neuroprotection, potentially preventing the progressive degeneration of dopaminergic neurons, which positions SUEHP as a minimal invasive therapeutic option for PD‐related pain.

**Nomenclature**
ACC: Anterior cingulate cortex
BBB: Blood–brain barrier
BDNF: Brain‐derived neurotrophic factor
BSA: Bovine serum albumin
CA: Cornu ammonis
CB1: Cannabinoid receptor 1
CBD: Cannabidiol
CD11b: Cluster of differentiation 11b
CNS: Central nervous system
DAB: 3,3′‐Diaminobenzidine
DAPI: 4′,6‐Diamidino‐2‐phenylindole
DBS: Deep brain stimulation
DEG: Differentially expressed gene
DG: Dentate gyrus
GABA: Gamma‐aminobutyric acid
GFAP: Glial fibrillary acidic protein
GSEA: Gene set enrichment analysis
JSOHP: Jungsongouhyul pharmacopuncture
MCH: Melanin‐concentrating hormone
MPTP: 1‐Methyl‐4‐phenyl‐1,2,3,6‐tetrahydropyridine
NeuN: Neuronal nuclei
PAG: Periaqueductal gray
PBS: Phosphate‐buffered saline
PCR: Polymerase chain reaction
PD: Parkinson’s disease
PPARγ: Peroxisome proliferator–activated receptor gamma
p‐p38: Phosphorylated p38 mitogen‐activated protein kinase
SEM: Standard error of the mean
SUEHP: SU‐Eohyeol pharmacopuncture
TH: Tyrosine hydroxylase
TX‐100: Triton X‐100

## Author Contributions

Jung Im Kim: writing–original manuscript, conceptualization, data curation, formal analysis, investigation, and methodology. Heerim Yeo: writing–original manuscript, data curation, formal analysis, and investigation. Yousang Jo: writing–original manuscript and formal analysis. Hyungjun Kim: writing–review and editing, conceptualization, funding acquisition, and supervision. Sang‐Min Park: writing–original manuscript, conceptualization, and investigation. No Soo Kim: writing–original manuscript, conceptualization, investigation, and supervision.

## Funding

This work was supported by Korea Institute of Oriental Medicine (KSN2224011).

## Disclosure

All authors read and approved the final manuscript for submission.

## Conflicts of Interest

The authors declare no conflicts of interest.

## Supporting Information

Additional supporting information can be found online in the Supporting Information section.

## Supporting information


**Supporting Information 1** Supporting Figure S1 shows the experimental schedule, MPTP‐induced pain induction, and dopaminergic neuronal death in the experimental mice.

**Supporting Information 2** Supporting Figure S2 shows astrocyte activation in the spinal cord of the experimental mice.

**Supporting Information 3** Supporting Figure S3 shows the motor function of the experimental mice as determined by the open field test.

**Supporting Information 4** Supporting Figure S4 shows the BDNF expression in the hippocampus of the experimental mice.

## Data Availability

The dataset supporting the conclusions of this article is included within the article and its additional files. The mRNA‐sequencing data for spinal cord and midbrain tissues are deposited and available in the GEO database with the identifier GSE302015.
