# Supplementary material for: SU‐Eohyeol Pharmacopuncture Ameliorates Parkinson’s Disease–Associated Pain via the CB1 and PPARγ Pathways in an MPTP‐Induced Mouse Model
Source: Pain Res Manag. 2026 May 31;2026:3334432. doi: 10.1155/prm/3334432 (PMC13239103; doi:10.1155/prm/3334432)

**Supplementary Figure S1.** Increased pain sensitivity and deficit of dopaminergic pathways in an MPTP-induced PD animal model. (a) Experimental timeline for assessing pain sensitivity in an MPTP-induced PD mouse model. The von Frey filament test was performed on the (b) left and (c) right hind paws, starting seven days prior to MPTP administration and on days 7, 10, 14, and 21 post-MPTP injection. Following behavioral assessments, brains were harvested and sectioned at a thickness of 20 μm to evaluate the expression of TH via DAB staining in the (d) SN and (f) striatum. (e) The number of TH-positive neurons in the SN and (g) the TH staining intensity in the striatum were measured using ImageJ. Scale bars: 200 μm (SN), 500 μm (striatum). Data are presented as mean ± SEM (n=6 for control, n=5 for MPTP group). ^a^*p*<0.05, ^aa^*p*<0.01, ^aaa^*p*<0.001 vs. Con. Abbreviations: BL, baseline; DAB, 3,3′-diaminobenzidine; MPTP, 1-methyl-4-phenyl-1,2,3,6-tetrahydropyridine; PD, Parkinson’s disease; TH, tyrosine hydroxylase; SEM, standard error of the mean; SN, substantia nigra; vF, von Frey filament test. Experimental groups: Con, saline treatment; MPTP, MPTP treatment.


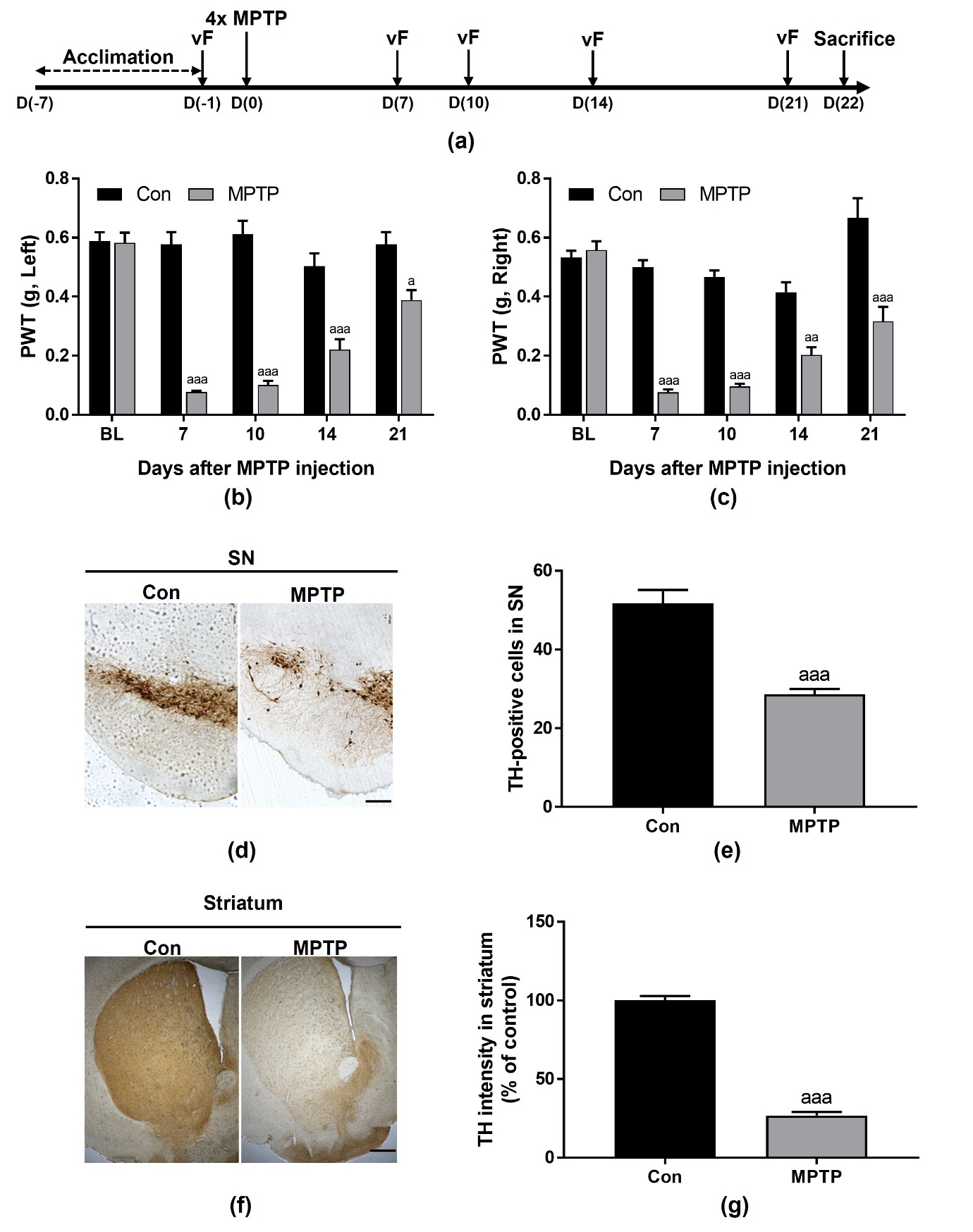

Supplement: Supplementary file 1 — Supporting Information 1 Supporting Figure S1 shows the experimental schedule, MPTP‐induced pain induction, and dopaminergic neuronal death in the experimental mice. [file PRM-2026-3334432-s001.docx]
