# Supplementary material for: SU‐Eohyeol Pharmacopuncture Ameliorates Parkinson’s Disease–Associated Pain via the CB1 and PPARγ Pathways in an MPTP‐Induced Mouse Model
Source: Pain Res Manag. 2026 May 31;2026:3334432. doi: 10.1155/prm/3334432 (PMC13239103; doi:10.1155/prm/3334432)

**Supplementary Figure S2.** Effect of SUEHP on astrocyte activation in the spinal cord of MPTP-induced PD animals. (a) Astrocyte activation was examined in the dorsal horn of the spinal cord (L3–L6) using immunofluorescence staining for GFAP. (b) Astrocyte activation was quantified by measuring the GFAP-stained area using ImageJ. Scale bars: 100 μm. Data are presented as mean ± SEM (n=5). *^a^p*<0.05 *vs.* Con. Abbreviations: DAPI, 4′,6-diamidino-2-phenylindole; GB34, acupoint “Yanglingquan”; GFAP, glial fibrillary acidic protein; MPTP, 1-methyl-4-phenyl-1,2,3,6-tetrahydropyridine; PD, Parkinson’s disease; SEM, standard error of the mean; SUEHP, SU-Eohyeol pharmacopuncture. Experimental groups: Con, saline control + saline injection at GB34; MPTP-Veh, MPTP + saline injection at GB34; MPTP-SU(GB), MPTP + SUEHP injection at GB34; MPTP-SU(NR), MPTP + SUEHP injection at non-relevant acupoint (gluteal region); MPTP-Ami, MPTP + amitriptyline treatment.

**Supplementary Figure S2**


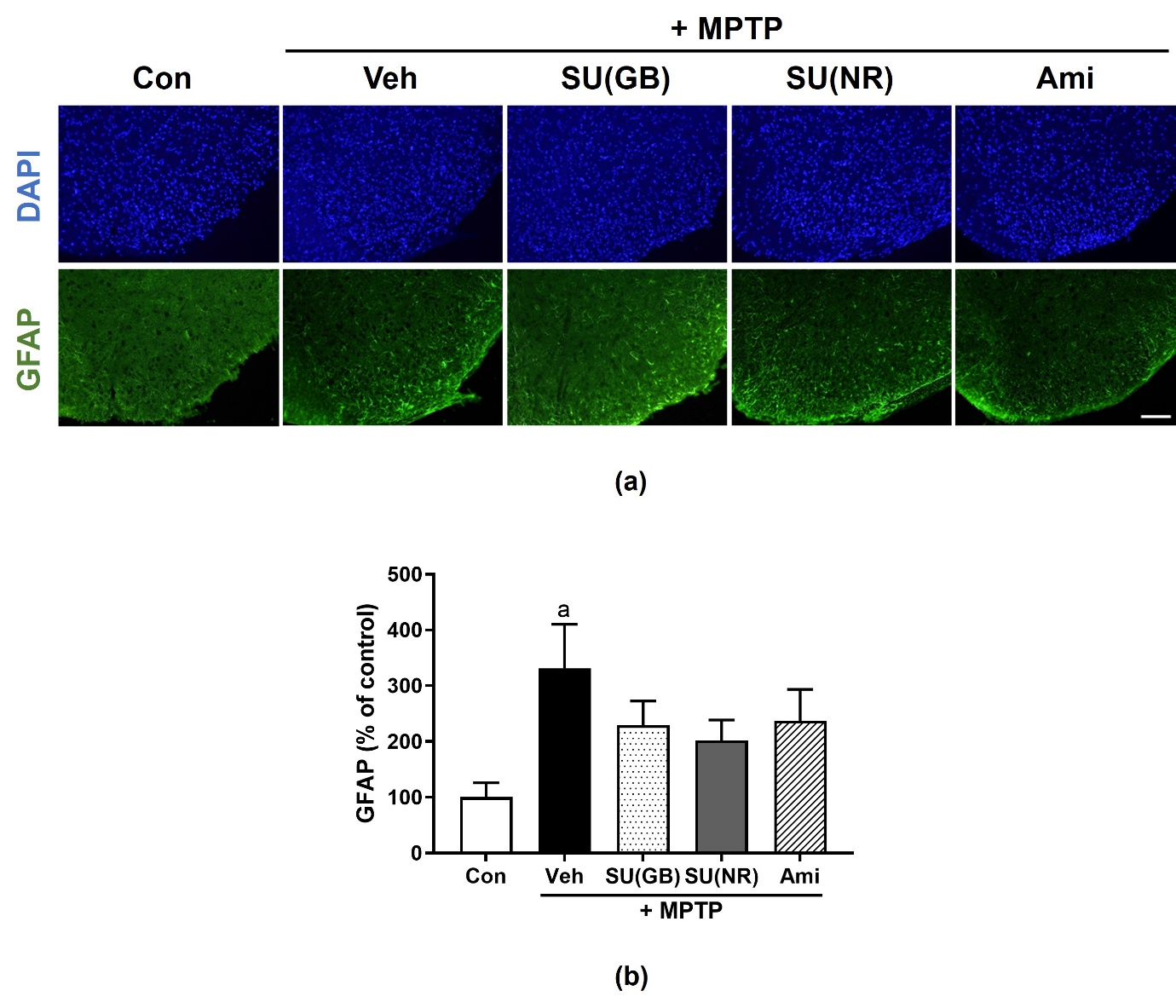

Supplement: Supplementary file 2 — Supporting Information 2 Supporting Figure S2 shows astrocyte activation in the spinal cord of the experimental mice. [file PRM-2026-3334432-s002.docx]
