# Supplementary material for: SU‐Eohyeol Pharmacopuncture Ameliorates Parkinson’s Disease–Associated Pain via the CB1 and PPARγ Pathways in an MPTP‐Induced Mouse Model
Source: Pain Res Manag. 2026 May 31;2026:3334432. doi: 10.1155/prm/3334432 (PMC13239103; doi:10.1155/prm/3334432)

**Supplementary Figure S3.** Effects of CB1 antagonist or PPARγ inhibitor treatment on the SUEHP-mediated recovery of motor function in the MPTP-induced PD animal model. (a) An open field test was performed to evaluate the therapeutic effect of SUEHP on motor dysfunction of PD animals and the roles of CB1- and PPARγ-mediated signaling pathways. (b) Total distance covered and (c) time spent in the center zone were analyzed. (d) Motor coordination of PD animals was determined by measuring the retention time using the rotarod test. Data are presented as the mean±SEM (n=10). ^a^*p*<0.05, ^aa^*p*<0.01, ^aaa^*p*<0.001 *vs.* Con. ^b^*p*<0.05, *vs.* MPTP-Veh. Abbreviations: CB1, cannabinoid receptor 1; GB34, acupoint “Yanglingquan”; MPTP, 1-methyl-4-phenyl-1,2,3,6-tetrahydropyridine; PD, Parkinson’s disease; PPARγ, peroxisome proliferator-activated receptor gamma; SEM, standard error of the mean; SUEHP, SU-Eohyeol pharmacopuncture. Experimental groups: Con, saline control + saline injection at GB34; MPTP-Veh, MPTP + saline injection at GB34; MPTP-SU, MPTP + SUEHP injection at GB34; MPTP-SU+S, MPTP + SUEHP injection at GB35 with SR141716A pretreatment; MPTP-SU+T, MPTP + SUEHP injection at GB34 with T0070907 pretreatment.

**Supplementary Figure S3**


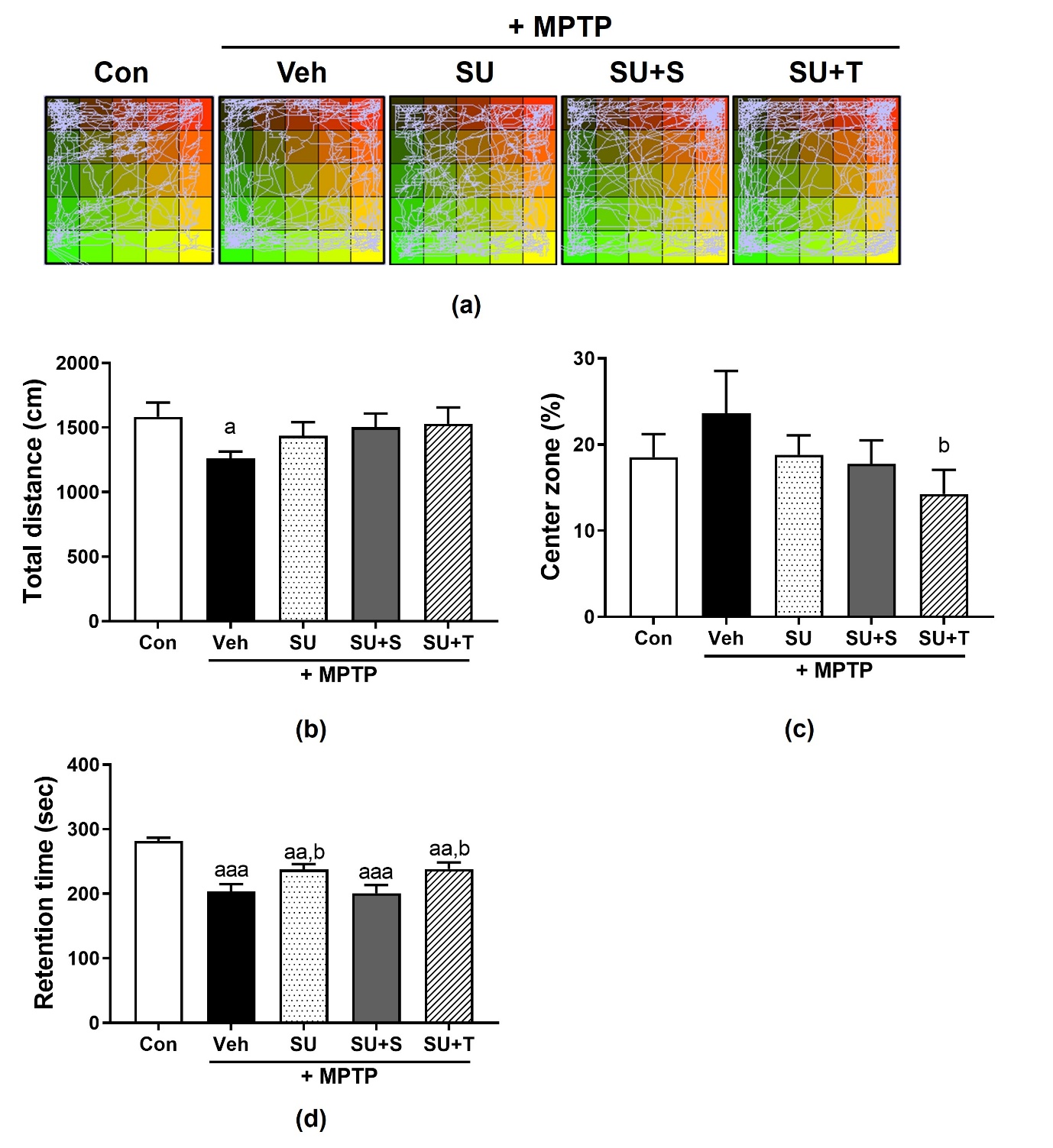

Supplement: Supplementary file 3 — Supporting Information 3 Supporting Figure S3 shows the motor function of the experimental mice as determined by the open field test. [file PRM-2026-3334432-s003.docx]
