# Supplementary material for: SU‐Eohyeol Pharmacopuncture Ameliorates Parkinson’s Disease–Associated Pain via the CB1 and PPARγ Pathways in an MPTP‐Induced Mouse Model
Source: Pain Res Manag. 2026 May 31;2026:3334432. doi: 10.1155/prm/3334432 (PMC13239103; doi:10.1155/prm/3334432)

**Supplementary Figure S4.** Effects of CB1 antagonist or PPARγ inhibitor treatment on the SUEHP-mediated recovery of BDNF expression in the hippocampus of the MPTP-induced PD animal model. BDNF expression in the hippocampus of MPTP-induced PD animals following SUEHP treatment at the GB34 acupoint in the presence of a CB1 antagonist (SR141716A) or PPARγ inhibitor (T0070907) co-treatment was determined using immunofluorescence staining. BDNF expression was analyzed in different hippocampal subregions, including (a, b) CA1, (c, d) CA3, and (e, f) the DG. Scale bar=100 μm. Data are presented as the mean±SEM (n=4). ^aaa^*p*<0.001 *vs.* Con. ^b^*p*<0.05, ^bb^p<0.01, ^bbb^*p*<0.001 *vs.* MPTP-Veh. Abbreviations: BDNF, brain-derived neurotrophic factor; CA, cornu ammonis; CB1, cannabinoid receptor 1; DAPI, 4′,6-diamidino-2-phenylindole; DG, dentate gyrus; GB34, acupoint “Yanglingquan”; MPTP, 1-methyl-4-phenyl-1,2,3,6-tetrahydropyridine; NeuN, neuronal nuclei; PD, Parkinson’s disease; PPARγ, peroxisome proliferator-activated receptor gamma; SEM, standard error of the mean; SUEHP, SU-Eohyeol pharmacopuncture. Experimental groups: Con, saline control + saline injection at GB34; MPTP-Veh, MPTP + saline injection at GB34; MPTP-SU, MPTP + SUEHP injection at GB34; MPTP-SU+S, MPTP + SUEHP injection at GB35 with SR141716A pretreatment; MPTP-SU+T, MPTP-SU+T, MPTP + SUEHP injection at GB34 with T0070907 pretreatment.

**Supplementary Figure S4**


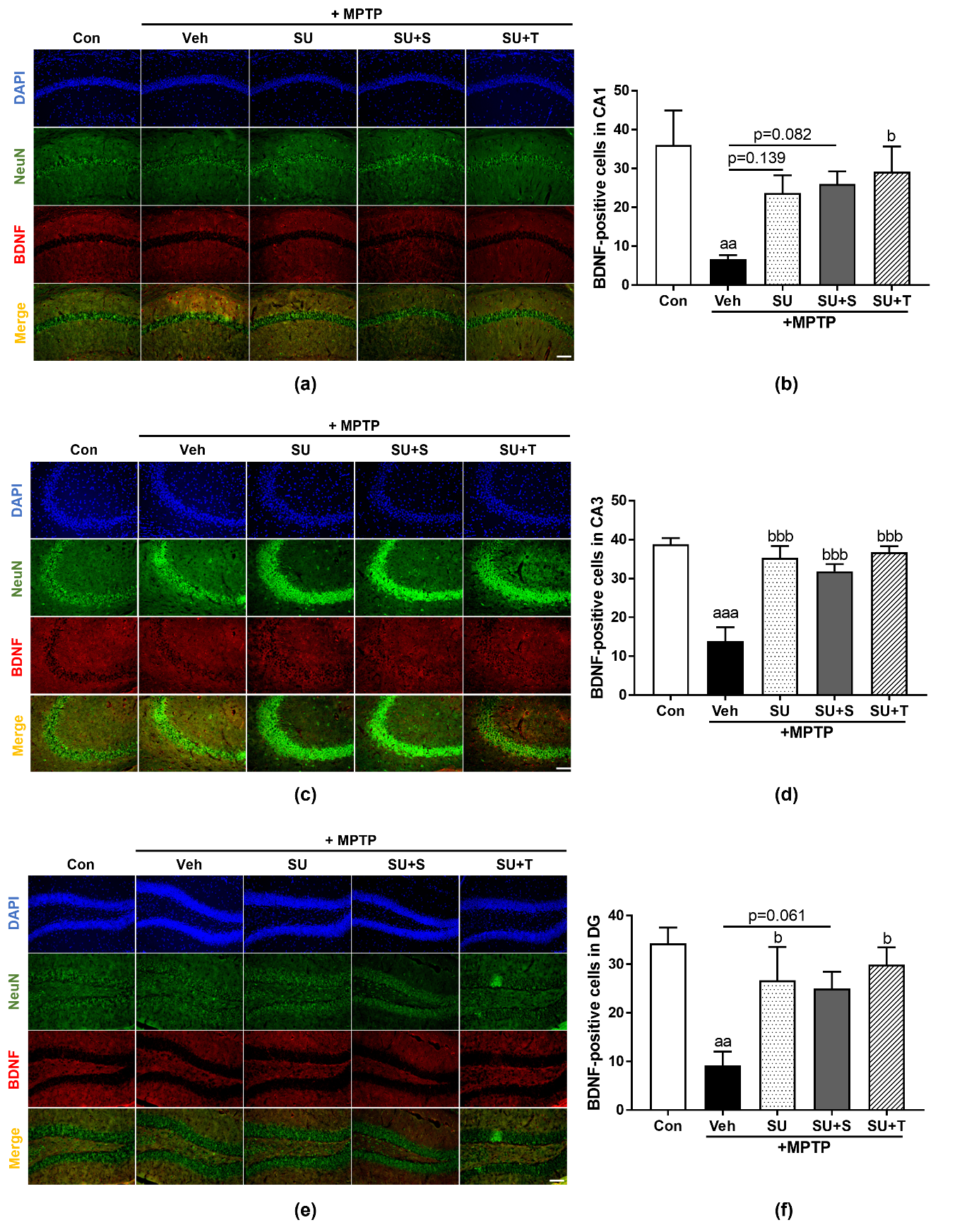

Supplement: Supplementary file 4 — Supporting Information 4 Supporting Figure S4 shows the BDNF expression in the hippocampus of the experimental mice. [file PRM-2026-3334432-s004.docx]
